# Identification of regional and cell type‐specific gene dysregulation in Progressive Supranuclear Palsy

**DOI:** 10.1002/alz.085494

**Published:** 2025-01-03

**Authors:** Jennifer A Grundman, Timothy S Chang, Riki Kawaguchi, Le Qi, Lawrence Chen, Kun Gao, Jing Ou, Jade Nguyen, Michael DeTure, Bailey Rawlinson, Clifton L. Dalgard, Huw R Morris, Daniel H. Geschwind

**Affiliations:** ^1^ University of California, Los Angeles, Los Angeles, CA USA; ^2^ Movement Disorders Programs, Department of Neurology, David Geffen School of Medicine, University of California, Los Angeles, Los Angeles, CA USA; ^3^ University of California, Los Angeles School of Medicine, Los Angeles, CA USA; ^4^ Mayo Clinic, Jacksonville, FL USA; ^5^ Uniformed Services University, Bethesda, MD USA; ^6^ University College London, London United Kingdom; ^7^ David Geffen School of Medicine, University of California, Los Angeles, Los Angeles, CA USA

## Abstract

**Background:**

Progressive supranuclear palsy (PSP) is a neurodegenerative disorder involving pathological deposition of tau that includes glial inclusions and specific regional vulnerability patterns. Therapeutic developments are hampered by incomplete understanding of disease mechanisms. Few studies have examined its cell type‐specific effects. We performed RNA‐sequencing in a large cohort of PSP patients and controls to understand molecular pathways disrupted across prefrontal cortex and caudate nucleus in PSP. We also performed multi‐omic single‐nucleus sequencing to deconvolute cell type‐specific changes.

**Method:**

RNA‐sequencing was conducted in 250 PSP patients and age‐ and sex‐matched controls, with a subset (N = 23) undergoing multi‐omic single‐nucleus RNA‐sequencing and ATAC‐sequencing (10x Genomics). Sequencing data was aligned to GRCh38 and quantified using STAR and RSEM (RNA‐seq) and CellRanger (single‐nucleus) and corrected for technical and biological covariates. Differential expression was calculated with Dream (RNA‐seq) and Dreamlet (single‐nucleus sequencing), and co‐expression network analysis (WGCNA) used to extract disease‐relevant gene modules.

**Result:**

RNA‐sequencing analysis identified ∼4000 differentially expressed genes in both brain regions. Despite differences in cellular composition and degree of disease impact, both regions share patterns of dysregulation related to protein folding and processing, indicating common pathological mechanisms. Differences between the regions includes disruption of cellular homeostasis and intracellular trafficking in caudate, while prefrontal cortex shows suppression of metabolic processes and immune response. Preliminary findings from the single cell genomic analyses suggest the greatest changes occur in neurons of both regions, where there is a shared pattern of protein processing dysregulation and a distinct enrichment in metabolic processing within prefrontal cortex interneurons.

**Conclusion:**

We identified thousands of dysregulated genes in two impacted brain regions in PSP and show that this dysregulation is reflected in specific neuronal populations in these regions. Identifying regional and specific cell type‐specific mechanisms related to PSP will further our understanding of causative disease mechanisms and provide new avenues for therapeutic developments.

